# Social Support as a Facilitator of Musical Self-Efficacy

**DOI:** 10.3389/fpsyg.2021.722082

**Published:** 2021-10-08

**Authors:** Santos Orejudo, Francisco Javier Zarza-Alzugaray, Oscar Casanova, Gary Edward McPherson

**Affiliations:** ^1^Faculty of Education, University of Zaragoza, Zaragoza, Spain; ^2^Melbourne Conservatorium of Music, University of Melbourne, Melbourne, VIC, Australia

**Keywords:** social support, musical self-efficacy, music students, academic level, age, parents, teachers

## Abstract

Previous research has shown that musical self-efficacy is one of the predictors of academic achievement, but few studies have analyzed the function of social support in the construction of musical self-efficacy. In this study we analyze the relationship between three sources of support perceived by music students – parents, teachers, and peers – and their influence on levels of self-efficacy for learning and for public performance. We analyze three groups of students under the hypothesis that relationships among those variables can vary with age and the level of education. A total of 444 students enrolled in six Spanish music schools, two music universities, and four advanced music schools, completed the Social Support Scale for Music Students, as well as the General Musical Self-Efficacy Scale. Results reveal significant relationships among the aforementioned variables, with considerable variation according to academic level. For the youngest students enrolled in advanced music schools (*conservatorios profesionales*), the role of parents and teachers was crucial, especially for predicting self-efficacy for learning, which, in turn, is the best predictor of self-efficacy for public performance. For the 16–18-year-olds enrolled in the same advanced music schools, their peers play a particularly relevant role in reinforcing their self-efficacy for learning. Social support had a negligible influence on the self-efficacy of university-level students, but they did experience a strong relationship between self-efficacy for learning, on the one hand, and public performance, on the other. We interpret these results in view of potential long-term careers in music, relating them with a series of different agents.

## Introduction

The theory of self-efficacy is one of the most relevant theoretical contributions to the study of human behavior. Originally defined by Bandura (1997, 2006), in his Socio-Cognitive Theory, self-efficacy is seen as “the conviction that one can successfully execute the behavior required to produce the outcome” (Bandura, 1997, p. 79). In other words, self-efficacy refers to the beliefs people hold about the extent to which they can complete a task in a particular situation: for example, in the area of music, which is the focus in this study (McPherson and McCormick, 2006). This approach to self-efficacy lends importance to the situational context and the specific domain in which we are analyzing a subject’s behavior, although other models can likewise be applied. Thus, for example, the Theory of General Self-Efficacy (Baessler and Schwarcer, 1996; Schwarzer and Jerusalem, 2010) postulates general self-efficacy as an attitude that can be adopted to face a series of stressors in a variety of different environments. Orejudo et al. (2017) have used that approach to define a profile of personal vulnerability in the face of performance anxiety within Barlow’s anxiety model (Barlow, 2000).

In both cases, the general approach and the specific focus are both relevant, since they each have the potential for explaining behaviors, cognitions, and emotional responses. A situational approach helps approximate performance in educational contexts (Zimmerman, 2000; Pajares and Schunk, 2001), leading to the postulation of the self-efficacy hypothesis (SEH), which has been used to help explain students’ choice of goals, the efforts they invest along with the strategies they employ to reach them, and the validation processes that serve as feedback for their study progress (Panadero and Alonso-Tapia, 2014). Music has been one of the concrete areas in which this theoretical framework has been developed and applied, as we expound below.

### Musical Self-Efficacy

Since the year 2000, McPherson and his collaborators started conducting studies on self-efficacy in the area of musical activity (McPherson and McCormick, 2000, 2006; McCormick and McPherson, 2003). Their first studies attempted to prove the relationship between self-efficacy and various levels of musical achievement. Subsequently, Papageorgi et al. (2007) postulated that self-efficacy is a key component in helping us to understand the training process undergone by music students, who, as they learn, need to develop skills to help them face a performance situation in front of an audience, and manage their performance anxiety. Along similar lines, Upitis et al. (2017c, p. 413) have described self-efficacy as “one’s beliefs in one’s abilities to achieve goals and complete tasks.” The tasks music students are required to accomplish are typically associated with performing in front of an audience: either in examinations, or in concerts. To be successful, one must have acquired the technical skills needed to prepare for and master repertoire to be performed. But apart from requiring the gradual mastery of those skills, musical training likewise necessitates the development of motivational abilities that enable the individual to persist in the task, especially when coping with difficulties and setbacks. In this way, self-efficacy is situated within models of self-regulated learning (Zimmerman, 2000; Varela et al., 2016).

Based on the above, it is evident that researchers who have studied the role played by self-efficacy in music training seek to understand: (1) the essential relationship between self-efficacy and musical performance; (2) the inclusion of self-efficacy in a theoretical framework that organizes data and allows researchers to structure their findings, making self-efficacy easy to evaluate; and (3) the particular types of factors that exert an influence on musical self-efficacy.

As mentioned above, one of the most relevant findings that can help us grasp the usefulness of self-efficacy in musical training is its relationship with public performance. Studying results from a sample of 332 music students, McCormick and McPherson (2003) reported that musical self-efficacy was the best predictor of their externally evaluated excellence in a public performance. Another study by the same team (McPherson and McCormick, 2006) replicated that result using an entirely different sample of 686 Australian students. Again, self-efficacy was shown to have a high predictive value for performance, thus confirming the significant role played by musical practice and by the cognitive strategies applied by the students. Subsequent studies, including Hewitt (2015) and Miksza and Tan (2015), have confirmed this relationship between self-efficacy and public performance.

Since self-efficacy is defined as a domain-specific construct, it can be adapted to other areas, such as music education (Hendricks, 2014). Several research teams have adapted a series of tools to two basic processes in that field: the study phase (preparation of repertoire during the phase of technical competency development), and the specific situation of performing in public in front of an audience. To examine both dimensions, Ritchie and Williamon (2007, 2011) devised the *General Musical Self-Efficacy Scale*, which was based on the *Self-Efficacy Scale* developed by Sherer and Adams (1983). By restricting itself to these two components, self-efficacy for learning, and self-efficacy for public performance, this model parsimoniously represents the most relevant tasks a student or a musician must accomplish to achieve a successful public performance.

Other authors have proposed additional areas of research in music. Thus, for instance, in a series of transcultural studies, Randles (Randles, 2011; Randles and Smith, 2012; Randles and Muhonen, 2015; Randles and Ballantyne, 2018; Randles and Tan, 2019) has analyzed aspects associated with creativity, composition, improvisation, and the act of playing different types of music. He considers that these dimensions of music serve as areas of development for musicians; areas in which to acquire a sense of competency in order to achieve satisfactory professional development. The results of these transcultural studies confirm the existence of the above-mentioned types of self-efficacy, while explaining certain differences among the levels achieved by students, based on their previous training experiences. Similarly, Watson (2010) has studied self-efficacy for improvising in jazz contexts, Egilmez (2015) for the handling of anxiety in one’s own perception of a public musical performance, and Miksza and Tan (2015) for self-regulated music learning. Further concrete areas of musical training can be studied with the construct of self-efficacy, since it defines itself as domain-specific. Kurtuldu and Bulut (2017) designed a scale to evaluate students’ self-efficacy toward piano lessons, and Watson (2010), adapted this scale for jazz. Other authors (Papageorgi et al., 2013; Girgin, 2020) have highlighted the lack of self-efficacy in facing a series of challenges and demands: a slump in motivation, on the one hand, or increased levels of burnout, on the other.

As mentioned above, studies in self-efficacy have benefited from the implementation of a wider theoretical framework regarding the subject of learning: concretely, self-regulated learning models (Zimmerman, 2000; Panadero and Alonso-Tapia, 2014). In studies in this area focusing on music, self-efficacy has also established itself as a key concept within self-regulated learning (McPherson and McCormick, 2000; Varela et al., 2016). An important way of explaining the relationship of self-efficacy with public music performance is by highlighting its role as a component of the self-regulated earning three-phase model (forethought, performance, and reflection) (McPherson, 2022). In this context, self-efficacy is seen as a component of self-motivation beliefs related to feelings of confidence, outcome expectations, interest, and passion which are based on the *want* (“I want to do this”) and *can* (“I can do this”) parts of motivation (McPherson, 2022; see also Varela et al., 2016). Self-efficacy includes the beliefs we hold about our own capacity to perform at an expected level of achievement (self-efficacy for performance), or the beliefs we hold about our own capacity to implement or learn the types of processes that will allow us to master a musical challenge within a practice session (self-efficacy for learning). We achieve our best level of performance when we feel confident, which is why self-efficacy beliefs are critical in expert performance. The emphasis is on believing we *can* do something (rather than *will* do something). Research shows that we tend to overestimate our capacity to achieve and perform. This is not necessarily bad, but our evaluations do need to be realistic because of this. Importantly, when our personal self-efficacy is high, we are more likely to set challenging goals for ourselves, and search for strategies to achieve them (McPherson, 2022).

The recent uptake in studies regarding self-regulated learning, the relative ease in evaluating it, and the existence of a well-defined theoretical corpus have given rise to a series of studies of the construct’s applicability (Hewitt, 2015; Miksza and Tan, 2015; Varela et al., 2016; Waters, 2020). Research has sought to improve students’ self-regulation through interventions (Watson, 2010; Mieder and Bugos, 2017; Miksza et al., 2018; MacAfee and Comeau, 2020), and studied the important role played by contextual factors in its development and modification, occasionally over brief periods of time (Hendricks, 2014), and using well-defined evaluation methods and validated measurement tools (Ritchie and Williamon, 2007, 2011; Watson, 2010; Randles and Muhonen, 2015; Kurtuldu and Bulut, 2017; McPherson et al., 2019; Osborne et al., 2020).

Although the number of studies on musical self-efficacy has increased in recent years, few studies have attempted to explore how it develops. In Bandura’s model, four different sources of self-efficacy are postulated: mastery experiences, vicarious experiences, verbal persuasion, and emotional states (Hendricks, 2014; Zelenak, 2015). The greatest amount of evidence has accumulated in relation with the importance of practice, especially the types of mastery experiences that are regarded as the basic condition for musical performance (McCormick and McPherson, 2003; McPherson and McCormick, 2006). However, not all approaches to practice are effective, since students need to simultaneously associate their practice routine with programing elements that are autonomous, strategic, and self-regulated; what researchers refer to as “deliberate practice” (Hallam et al., 2012; McPherson et al., 2012; Upitis et al., 2017c). This kind of practice approach increases with the passage of time under the influence of academic requirements that progressively become more demanding.

Further information has been gathered about the effect of emotional states, mostly associated with performance anxiety (Papageorgi et al., 2010). Hardly any evidence has been gathered, however, regarding the eventual influence of other sources. Although clear evidence has been found for the influence exerted by parents, teachers and peers on the development of a musical career (Orejudo et al., 2020), practically no evidence has been gathered to ascertain whether the role played by these agents leads to an improvement in musical self-efficacy. Previous studies have shown the role they can play by strengthening other elements associated with success in a musical career, such as providing the trainee with a series of resources to help them handle performance anxiety (Zarza-Alzugaray et al., 2020), but no direct data has been provided regarding the relationship between the support coming from those agents and the development of the musical self-efficacy. Nevertheless, before we continue to address this study’s goals, we proceed to analyze in further detail the role of social support in musical training.

### Social Support in Music

As shown by Gruber et al. (2008), and Lehmann and Kristensen (2014) parents, teachers, and peers provide an important source of information for learners because of their role as “persons in the shadow.” Successful musical careers always rely on the existence of certain people who are relevant and key in supporting the artist’s training, in early as well as in advanced stages. Here we are dealing with the psychological concept of “social support” (Caplan, 1974; Cobb, 1976; Sarason et al., 1990). Social support involves different forms of psychological support and resources provided by significant people in the learner’s environment who help satisfy their basic needs in interacting with others, act as a source that can be trusted, and are valued and loved because they are able to maintain open communication channels that are based on mutual feelings of responsibility and commitment.

Growing evidence demonstrates the presence of social support as a key factor that determines musical success (Davidson et al., 1996; McPherson and Davidson, 2002; Creech and Hallam, 2003; Sichivitsa, 2007; Margiotta, 2011; Nogaj and Ossowski, 2015). Moore et al. (2003) related social support to progress in an artist’s musical career; Nogaj and Ossowski (2015) related it to achievement, Sichivitsa (2007) found that parental support is a basic factor in music students’ self-concept, whereas Howe and Sloboda (1991) highlight the role played by parents and siblings in the initial stages of musical training. Social support is defined as a multidimensional construct that involves different types of support: instrumental and emotional support, as well as what a series of personal agents can provide – in the case of music, those agents are the family, teachers, and peers (Orejudo et al., 2020). Creech (2009) has specified that parents support their children by three different types of means: behavioral support, cognitive support, and personal support. Through these types of support, parents can enhance the teacher’s educational task by helping the student organize their study, providing them with opportunities to interact with music, and helping them establish expectations and goals. As can be readily observed, such parental activities come to form part of a series of conditions that encourage the student’s development of positive perceptions about their own personal value, thereby generating self-regulated learning skills.

Although parents undoubtedly fulfill a basic role in their children’s musical training, this does not occur at the margins of what is achieved by teachers and peers. All three groups are jointly regarded as the main support sources for music students, responsible for generating the motivational and emotional processes they need in order to pursue their training career (Ryan et al., 2000; Lehmann and Kristensen, 2014; Nogaj and Ossowski, 2015). Indeed, these three “source groups” are not regarded as mutually independent. For instance, parents who have enjoyed musical training and have a direct relationship with music are generally perceived as better sources of support than those for whom it is not the case (Sichivitsa, 2007; Ritchie and Williamon, 2013; Orejudo et al., 2020). They effectively encourage the student to persevere with their musical training (Jeppsson and Lindgren, 2018). Upitis et al. (2017a) found that a family relative or a custodian who plays an instrument provides a positive contribution to self-efficacy. The student has a greater enjoyment of the training situation, and this, in turn, can reinforce intrinsic motivation that might otherwise be lacking.

Teachers are further key agents in music student training. By establishing a direct relationship with self-efficacy sources, teachers can play a key role in the learning process, thereby providing an essential contribution to student motivation (Upitis et al., 2017c). This activity involves a number of aspects such as the establishment of short-term and long-term goals, monitoring the latter process, choosing repertoire, providing feedback *via* exams or a general evaluation of the student, teaching coping strategies to face performance anxiety, and acting as a social and emotional support agent in collaboration with the family. Regarding this important role played by teachers, there is a certain amount of evidence. Upitis et al. (2017a) report that teacher quality is an important factor enabling students to enjoy their achievements, particularly their public performances. Waters (2020) examined which factors have a decisive influence on the effectiveness of orientation provided by the teachers: students put their teachers’ advice to best use when they approach the learning context with a proactive attitude. Conversely, when students are not sufficiently autonomous in this sense, those who manage to adopt the strategies suggested by the teachers nevertheless have very little perception of how to control them and thereby do not succeed in transforming them into tools that improve their learning. Such students eventually perceive that they have less control in shaping their learning, with the result that their self-efficacy declines.

Peers are likewise regarded as a source of social support for musicians, but little evidence has been found of their relationship with musical development. Hendricks (2014) ascertained that when girls, in particular, feel that they are receiving a substantial amount of social support, they experience greater levels of self-efficacy. This is more likely to occur if the context is not perceived as being highly competitive. Siblings can also be a source of motivation for music students. Howe and Sloboda (1991) ascertained that elder brothers and sisters play an important role in the musical practice activities of their younger siblings.

### Aims, Research Questions, and Objectives

As mentioned above, in recent years a considerable number of studies have been published, ascertaining that self-efficacy is a useful construct for the analysis of musical training. A series of studies have proved its relationship with performance in different contexts, and/or have developed tools to evaluate it in such contexts, giving rise, on occasion, to full-fledged intervention programs. But few studies exist on the role of social support in maintaining musical self-efficacy. Age, sex, and type of instrument (solo or orchestral) are relevant variables analyzing musical self-efficacy as well as performance anxiety (Casanova et al., 2018; Zarza-Alzugaray et al., 2020). In this study, however, we have focused on the variables of age and formative level, since they have been less analyzed until now. To our knowledge, no study has been published with an attempt to compare the sole exerted in social support by different sources – parents, teachers, and peers – at different levels of education.

Our study’s purpose was thus to analyze the types and amount of support perceived by music students at two academic levels: those enrolled in university-level music academies (*conservatorios superiores*), and those enrolled in advanced music schools (*conservatorios profesionales*). *Conservatorios superiores* are institutions of musical learning for students who want to embark on a professional music-related career. At the same age at which they would start studying at university, they gain access to *conservatorios superiores* after having concluded studies in a *conservatorio professional.* This is usually when they are 18 years old. At the other educational level, *conservatorios profesionales* enroll students with more heterogeneous profiles: some are studying to gain access to a *conservatorio superior*, while others are learning music without necessarily holding a long-term professional perspective in mind. For purposes of analysis, we differentiate two age groups in *conservatorios profesionales*: younger students, on the one hand, and 16–18-year-olds, on the other. We chose to apply this division based on evidence (Orejudo et al., 2020) that students in the latter age group are combining musical training with secondary education which allows them access to university, and are going through a decision-making process regarding their professional future, which can affect the level of their commitment to musical activities.

We expected to find a relationship between social support and self-efficacy for learning and for public performance (Ritchie and Williamon, 2007, 2011; Upitis et al., 2017c), given the possibility that such relationships can vary in terms of age and academic level. We therefore carried out the analysis on three separate groups of students: university-level music students (*conservatorios superiores*), 16–18-year-old students enrolled in *conservatorios profesionales*, and younger students (11–15-year-old) enrolled in the latter kind of institution. In support of this assumption, certain authors postulate that family support should be more relevant in early stages (Davidson et al., 1996; Margiotta, 2011), and that teachers in initial musical training stages need to have a series of competencies that differ from those required for more advanced stages (Moore et al., 2003).

A second assumption refers to the relationship among the different types of self-efficacy. We speculated that self-efficacy for learning will be a strong predictor of self-efficacy for public performance. This seems plausible within the theoretical framework of self-regulated learning, in which the preparation of repertoire prior to performance and the management of performance anxiety serves as relevant factors in the development of musical competency. We thus expected to find a direct relationship between these, although, admittedly, learning situations do not necessarily imply performance experiences; the predictors of the two types of self-efficacy might thus eventually be different.

To test these assumptions and to ascertain whether the relationships among these variables can differ in function of age, we analyzed our data using structural equation modeling (SEM) with the sources of support (parents, teachers, and peers) as exogenous variables and the sources of self-efficacy as endogenous variables. We assumed that the relationship among them could be direct, and that self-efficacy would have a relationship of partial mediation with the sources of support. This analysis technique also allowed us to compare equality of regression weights in different groups (Byrne, 2010).

## Materials and Methods

### Participants

Our sample comprised 415 music students, 296 of whom (71.3%) were enrolled in *conservatorios profesionales* (advanced, pre-university music schools), whereas 119 (28.7%) were enrolled in *conservatorios superiores* (university-level music academies). We established three large age groups: 141 [34%, *Mean age* (*M*) = 13.69, SD = 1.17, range: 11–15] participants were 15 years old or younger (enrolled in *conservatorios profesionales*); 158 were ages 16–18 (38%, *M* = 16.91, SD = 0.80), all enrolled in *conservatorios profesionales*; the last group, age 19 and older (28%, *M* = 22.41, SD = 4.45, 19–43), were enrolled in *conservatorios superiores* (university-level music academies). Regarding distribution by gender, 44.6% (*n* = 185) were male, whereas 55.4% (*n* = 230) were female, without any significant association (χ^2^ = 4.194; *p* = 0.123) between a student’s gender and their age group.

### Measures

The *Social Support Scale* proposed by Ryan et al. (2000) is designed to evaluate the level of social support perceived by music students. It measures that support through a series of independent scales corresponding to each of the social agents: parents, teachers, and peers, associated with 12, 9, and 10 items respectively, measured on a 7-point Likert-type scale (from 1, “not very much,” to 7, “a lot”). A Spanish version of this scale (Orejudo et al., 2020) has been validated for the academic levels featured in this study. The authors found one factorial structure for parent support (9 items, α = 0.866) and teacher support (10 items, α = 0.866), but two different factors for peer support: one related to musical training activities (5 items, α = 0.785), and the other related to facing taunts (3 items, α = 0.935). For this reason, we used the same four social support subscales in our study.

The *General Musical Self-Efficacy Scale* proposed by Ritchie and Williamon (2007, 2011) is a 1–7-point Likert scale (completely disagree-completely agree) made up of 22 items grouped into two subscales: musical self-efficacy for learning, and musical self-efficacy for performing. Six items in each of the subscales are reverse-coded: items 2, 4, 5, 8, 10, and 11 in the learning factor, and items 2, 3, 4, 6, 7, and 8 in the performance factor. In its English-language version, this scale has good psychometric properties as applied to different age groups, including young students (Ritchie and Williamon, 2013). A Spanish version (Orejudo et al., 2020) confirms the scale’s reliability and validity. Internal consistency was good both in the “self-efficacy for learning” scale (10 items, α = 0.773) as well as in the “self-efficacy for performance” scale (10 items, α = 0.773). There was also good temporal stability (correlation ranging from 0.515 to 0.539 after a period of 1 month).

### Procedure

After having received an affirmative response from the above-cited institutions of musical learning, we proceeded to gather the data in person, on the premises of each institution. The research team or a local professor visited the academies in order to operate *in situ*, with the task of administering and gathering the questionnaires (this lasted approximately 30 min per session). Students participated on a voluntary, anonymous basis, and they had no external incentive to participate in the study.

### Statistical Procedure

**FIGURE 1:**
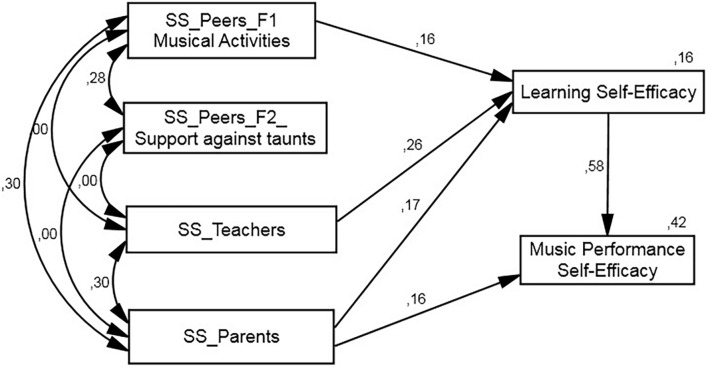
Up to 15 years old graph path.

**FIGURE 2:**
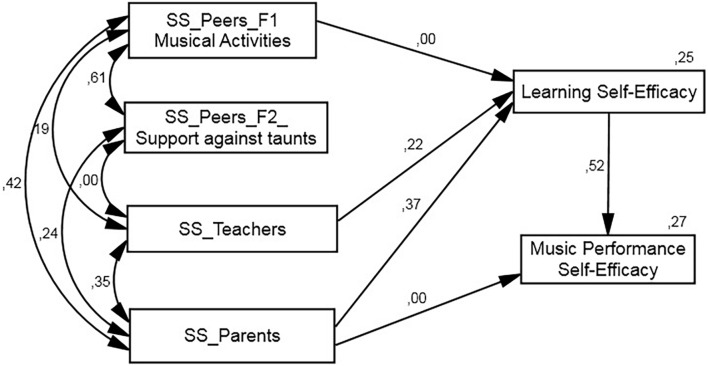
Between 16 to 18 years old graph path.

**FIGURE 3:**
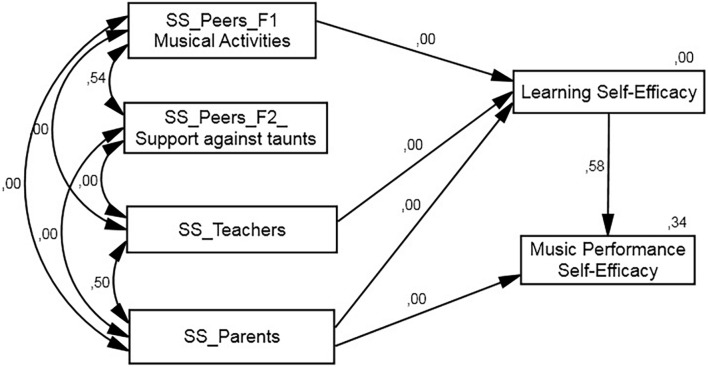
From 19 years old graph path.

Analysis of results was conducted in two phases. In a first phase, we applied descriptive analysis of the means of the scales that were used, differentiating by age group to conduct an initial exploration of results and to test whether there were differences. Correlations were obtained among all the factor scores of the variables in the three age-groups. In view of the high number of correlations in each group, we adjusted the level of significance by applying the Bonferroni correction (*p* = 0.0033). In a second phase, we tested the hypothetical model of causal structure by applying *SEM*. In this model we posited the Social Support Scales of parents, teachers, and peers as exogenous variables, and the two self-efficacy subscales as endogenous variables. The model is displayed in Figures 1–3, and was tested with IBM-SPSS software and its AMOS extension (v. 17). The estimation method chosen to test the measurement model was maximum likelihood whenever multivariate normal distribution criteria were met. We initially obtained correlations among all exogenous and endogenous variables in each of the subsamples we analyzed. Then a comparison was made between the two subsamples by applying Fisher’s Z transformation of the correlation coefficient. The model’s goodness of fit was tested using the χ^2^ test, as well as the normal and the χ^2^ degrees of freedom ratio (*DCIM/GL* in Amos), by *RMSEA* and *GFI* indicators, and by their critical levels as indicated by authors such as Schermelleh-Engel et al. (2003) and Byrne (2010). We applied multi-group analysis to verify whether the interviewees of different age groups displayed significant differences in terms of influencing relationships. To make this distinction between models, we compared a series of nested models, the results of which are described in section “Results.” To contrast differences between groups, the models were compared by calculating differences in χ^2^ and the *AIC* index (Byrne, 2010).

## Results

An initial result (Table 1) shows significant differences (*p* < 0.05) between the three age groups in all posited variables, except for F2, the factor of “peer social support in the face of taunts” (*F_2__–__412_* = 1.022; *p* = 0.361), although the size effect reported by the η^2^ is admittedly small. Comparing the group of youngest participants (<15 years old) with the aged 16–18, the *post hoc* tests reveal significant mean differences in the factors of self-efficacy for learning (*p* = 0.001) in parental social support (*p* = 0.004), and in teacher support (*p* = 0.020), with higher values in the younger groups (Table 1). In the factor of self-efficacy for public performance, the group of 16–18-year-olds scores lower than the two other groups (*p* < 0.05). Finally, in the F1 factor of “peer social support for musical learning,” the oldest group of students (>19 years old) scores higher than the two other age groups, whereby the highest mean is in the oldest group (*p* < 0.05).

**TABLE 1:** ANOVA self-efficacy and social support × age – level.

**Variable**	**Age–level**	***N***	**Mean**	**Standard deviation**	***F***	**Significant**	**η^2^**
Learning self-efficacy	≤15 years old secondary	141	58.66	7.01	6.997	0.001	0.033
	16–18 years old secondary	158	55.51	7.89			
	≥19 tertiary	116	57.40	6.98			
	Total	415	57.11	7.46			
Music performance self-efficacy	≤15 secondary	141	52.94	9.19	7.853	0.000	0.037
	16–18 secondary	158	49.70	8.52			
	≥19 tertiary	116	53.40	8.12			
	Total	415	51.84	8.79			
Parents social support	≤15 secondary	141	56.10	5.90	4.685	0.010	0.022
	16–18 secondary	158	53.63	7.75			
	≥19 tertiary	116	55.42	7.83			
	Total	415	54.97	7.26			
Teachers social support	≤15 secondary	141	50.49	7.87	5.489	0.012	0.021
	16–18 secondary	158	47.59	10.04			
	≥19 tertiary	116	50.21	9.40			
	Total	415	49.31	9.25			
Peers social support F1	≤15 secondary	141	23.83	6.62	5.797	0.003	0.027
	16–18 secondary	158	24.41	5.65			
	≥19 tertiary	116	26.37	6.31			
	Total	415	24.76	6.25			
Peers social support F2	≤15 secondary	141	15.91	5.73	1.022	0.361	0.005
	16–18 secondary	158	15.35	5.03			
	≥19 tertiary	116	14.96	5.32			
	Total	415	15.43	5.36			

*Peers social support F1: peers support musical activities. Peers social support F2: peers support face of taunts.*

As shown in Table 1, a series of significant correlations can be observed in the three age groups among the factors posited for this study. The factors we attempt to explain by social support display significant relationships (*p* < 0.003) with social support coming from parents and teachers, particularly in the group of high-school-age students. Neither of the two older groups of students fulfills the established significance criterion. Differences appear in function of age groups. Parent support and self-efficacy for learning are more pronounced in the youngest group of students (*r* = 0.455) than in the 16–18-year-olds (*r* = 0.297) and in those over 19 (*r* = 0.271). In the case of peers, there is a strong correlation between the two types of support, but in neither case do they exceed the limit established to consider the correlation as significant with self-efficacy, despite the values that appear in the intermediate age group (*r* = 0.220, *p* = 0.007). Correlations between self-efficacy for learning and self-efficacy for public performance are high in all age groups (*r* = 0.553–0.624), and there are no statistically significant differences among groups.

Table 2 also displays the correlations we observed between age groups and sources of support. Most correlations, although significant with *p* ≤ 0.05, are no longer significant with the Bonferroni correction. Parental support correlates highly with teacher support, and peer support with musical activities. Magnitude of correlation is average, except, curiously, the correlation among parents and teachers in the oldest age group, which is greater (*r* = 0.505 vs. *r* = 0.360 and *r* = 0.308) than in the two younger groups. Conversely, parent support in the youngest group is associated with peer support for musical activities (*r* = 0.429 vs. *r* = 0.306 and *r* = 0.207). As was to be expected, the highest correlations can be found between peer support for musical activities and facing taunts, although the correlation in the 16–18-year-old group (*r* = 0.278) is much lower than in the youngest group (*r* = 0.611) and the oldest group (*r* = 0.541). Applying the Bonferroni correction, parent support and teacher support do not correlate with peer support.

**TABLE 2:** Correlations.

		**Secondary level**		**Secondary level**		**Tertiary level**	
		**≤15 years old**		**16–18 years old**		**≥19 years old**	
		***r***	**Significant**	***r***	**Significant**	***r***	**Significant**
**Music learning self-efficacy**							
Parents SS		0.455	<0.001	0.297	<0.001	0.271	0.005
Teachers SS		0.349	<0.001	0.321	<0.001	0.275	0.004
Peers SS F1		0.197	0.022	0.220	0.007	0.196	0.039
Peers SS F2		0.109	0.199	0.123	0.125	0.078	0.404
**Music performance self-efficacy**							
Learning self-efficacy		0.553	<0.001	0.587	<0.001	0.622	<0.001
Parents SS		0.374	<0.001	0.352	<0.001	0.284	0.003
Teachers SS		0.291	<0.001	0.199	0.015	0.272	0.005
Peers SS F1		0.195	0.023	0.092	0.249	0.071	0.447
Peers SS F2		0.087	0.307	0.019	0.809	−0.070	0.454
**Sources of social support**							
Parents	Teachers	0.360	<0.001	0.308	<0.001	0.505	<0.001
Peers F1	Parents	0.429	<0.001	0.306	<0.001	0.277	0.004
Peers F1	Teachers	0.205	0.018	0.030	0.705	0.162	0.086
Peers F1	Peers F2	0.611	<0.001	0.278	<0.001	0.541	<0.001
Peers F2	Parents	0.253	0.004	−0.006	0.939	0.198	0.037
Peers F2	Teachers	0.032	0.709	0.053	0.511	0.123	0.19

*Peers social support F1: peer support for musical activities. Peers social support F2: peer support in the face of taunts.*

The *SEM* model, constructed according to our postulated theoretical model (Figure 1), has optimal fit (Table 3), not only when the regression parameters are set equally for all three groups (structural weights model), but also when restrictions are introduced (unconstrained model). The unconstrained model nevertheless indicates that certain relationships established in the model have non-significant values. We therefore postulated a new model: structural weights without non-significant weights, in which those parameters are set at zero, whereby the remaining values are kept the same in all groups. On this model, which displays adequate fit values (χ^2^ = 38.535, *gl* = 22, *p* = 0.016, χ^2^*/gl* = 1.752, *CFI* = 0.0966, *RMSEA* = 0.043), we tested the hypothesis that regression weight could be different in some groups. To ascertain this, we freed up each parameter in order to ascertain whether the new model improved the former one’s fit. Table 3 displays the different options we tested. One of the models improves the previous model’s fit (Δχ^2^ = 5.371, *gl* = 1, *p* = 0.020, Δ_AIC_ = 3.371), and establishes that the regression weights of b7 (social support of parents for self-efficacy in learning) are different.

**TABLE 3:** Results of the *SEM* model.

**Model**	**χ^2^**	**DF**	**Significant**	**CMIN/DF**	***RMSEA***	***CFI***	**TLI**	**Akaike**
Unconstrained	1.256	3	0.74	0.419	1.000	1.000	0.00	157.256
Structural weights	18.596	19	0.483	0.979	1.000	1.000	0.00	142.596
Structural weights–without n.s.	38.535	22	0.016	1.752	0.966	0.966	0.043	156.535
Free_b8_1	37.824	21	0.014	1.801	0.965	0.965	0.044	157.824
Free_b8_2	35.959	21	0.022	1.712	0.969	0.969	0.042	155.959
Free_b8_3	37.679	21	0.014	1.794	0.965	0.965	0.044	157.679
b6_1 and b6_2_free	38.178	21	0.012	1.818	0.964	0.964	0.045	158.178
b7_1 and b7_2_free	33.164	21	0.044	1.579	0.975	0.975	0.037	153.164

*b8_1 = learning self-efficacy to music performance self-efficacy: group secondary level, ≤15 years old. b8_2 = learning self-efficacy to music performance self-efficacy: group secondary level, 16–18 years old. b8_3 = learning self-efficacy to music performance self-efficacy: group tertiary level, ≥19 years old. b6_1 and b6_2_free: teachers social support to learning self-efficacy groups 1 and 2. b7_1 and b7_2_free: parents social support to learning self-efficacy, groups 1 and 2.*

As Figure 1 shows, teacher support (β = 0.22) and parent support (β = 0.37) in the group of youngest students was directly related with self-efficacy for learning, which, in turn, significantly mediates (β = 0.52) self-efficacy for music performance and explains 33.2% of its variance. In the case of self-efficacy for learning, self-efficacy for music performance explained 25% of its variance. In this mediation model, it is important to note that parent support (β = 0.193) and teacher support (β = 0.116) provided an indirect contribution to self-efficacy in public performance.

In the group of 16–18-year-olds (Figure 2), self-efficacy for learning was again related to teacher support (β = 0.26), but parents now had lower mediation than in the youngest group (β = 0.17). Although there are two differences compared to the model of the youngest group, peer support was related to self-efficacy for learning (β = 0.16), and explained 17% of that variable. Parents, for their part, had a direct influence on self-efficacy for public performance (β = 0.16), and this, along with self-efficacy for learning (β = 0.58), helped explain 42% of self-efficacy for public performance. Once again, one can note indirect effects of teacher support (β = 0.149), of parent support (β = 0.099), and of peer support (β = 0.094) on self-efficacy for interpretation. The total effect of parent support on public performance reached a total of β = 0.263.

None of the support sources provided a significant contribution to the model in the oldest group (Figure 3). The only relation that can be observe was that self-efficacy for learning predicted self-efficacy for public performance (β = 0.58), explaining 34% of the variance.

## Discussion and Conclusion

This study was designed to examine the relationships between social support perceived by music students and their self-efficacy for learning, as well as for facing performance situations, using students of different ages and academic levels. We analyzed relationships within an *SEM* model in which support sources were the exogenous variables, and the two endogenous variables were self-efficacy for learning and self-efficacy for public performance, with a relationship of mediation between them. Results provide clear evidenced of an important relationship between self-efficacy for learning and self-efficacy for public performance in all three age groups. This result is especially relevant for the oldest group, where self-efficacy for learning is the sole predictor of self-efficacy for public performance. Our data have evidenced a relationship between social support and self-efficacy, but only for students in the two younger age groups. In other words, for older students, who have more experience, there is no evidence that social support effects self-efficacy.

These findings are important from the perspective of music education. Although previous studies have highlighted the importance of parents, teachers, and peers in students’ musical training (Moore et al., 2003; McPherson, 2009; Lehmann and Kristensen, 2014), none have yet tested the relation between sources of support and one of the self-regulated learning model’s main variables which has the closest relation with musical practice: self-efficacy (McPherson and Zimmerman, 2011; Varela et al., 2016), neither has the assumption been tested on different age groups and academic levels. At the same time, our study provides new evidence of the importance of social support in the development of a musical career and is in accord with previous studies that highlighted the importance of parents in the musical education of their children. McPherson (2009) postulated that such support needs to be integrated into a framework that equips the student with a wide array of strategies to help them meet the demands of a musical career and make progress therein: the framework of self-regulated learning. However, little evidence on these seemingly critical aspects has been gathered until now.

More concretely, we observed that parental support of secondary school students became the main predictor of self-efficacy (since in this study we did not gather responses from students over 18 years old enrolled in *conservatorios profesionales*). As mentioned above, little evidence had been previously gathered regarding the relationship between social support and self-efficacy, either as an influence on musical learning or on public performance. Other studies, however, have dealt with the importance of social support in early musical training stages (Howe and Sloboda, 1991; Davidson et al., 1996; McPherson and Davidson, 2002; Moore et al., 2003; Sichivitsa, 2007; Margiotta, 2011). More recently, Upitis et al. (2017b) demonstrated the importance of the involvement of families in their children’s musical progress, from initial musical training to adolescence. They highlight day-to-day activities carried out by the parents to help their children’s progress: study at home, setting weekly or yearly goals, providing instrumental support, contacting teachers, and teaching concrete strategies. Those authors likewise observed how parents gradually reduce the amount of support they provide as students grow older and become more autonomous. The same study showed that parents as well as teachers are sources of support (MacMillan, 2004; Creech, 2009). Given the importance generally attributed to the family as a source of support, this gives rise to a new debate over family variables that can affect support levels. Future studies could explore variables such as: family expectations or family beliefs about what consists in the necessary amount of practice, parental ability to help the child go on practicing, or the relationship of parents and other family members with music. McPherson and Davidson (2002) evidenced that mothers of 7–9-year-olds who were initiating musical training and who had more ability to determine the amount of support their children needed in order to practice on a regular basis could indeed increase their offspring’s possibilities of pursuing training. In older students, Orejudo et al. (2020) found that the parents’ relationship with music, either as professional musicians or as music teachers, is an important predictor of support as perceived by their children in the course of their musical training. However, it is important to point out that support provided by families can be quite different according to training level, as our study has revealed. Thus, McPherson and Davidson (2002) ascertained that parent support for 7–8-year-old children who are in initial training impinges decisively on whether those children will continue or not. This factor can be key, but not indispensable, for the pursual of a musical career, as evidenced by Howe and Sloboda (1991), who found that children of that age had a high degree of motivation that did not necessarily stem from a family environment that was closely associated with music.

Thus, the research presented here adds the importance of teacher social support to that of the family as a learning resource throughout musical training at secondary-school age. As noted above, few studies have been published on self-efficacy, but other papers do demonstrate its role in musical training (Moore et al., 2003). Upitis et al. (2017b) highlight the importance of the quality of teachers as the main factor that promotes student progress at that academic level. The study was carried out from the family perspective, but it is likewise corroborated from the vantage point of the students (Upitis et al., 2017c). As in the case of families, it is important to be able to analyze the tasks carried out by teachers that help students develop their self-efficacy. An analysis of the tasks involved in teacher support (Ryan et al., 2000) confirms that the involved factors are associated with the creation of a positive atmosphere in the classroom (questionnaire items: “makes music class interesting,” “teaches music you like,” “often gives you a chance to choose what musical activities you do,” “wants you to try your best and not worry if you make mistakes”), but are likewise associated with the teachers’ positive expectations of what their students can accomplish (“wants you to pass music exams,” “thinks you could have a job in music when you get older”), with the way they value them globally as musicians [“thinks you are good at playing an instrument,” “praises you (tells you ‘well done’) for the work you do in music class”] or with the way they deal with mistakes. In their daily encounters with teachers, students expect that the latter should become sources of support for them, not only in the area of learning, but also in terms of more global aspects of their wellbeing such as helping them learn to deal with the stressors involved in musical training (Perkins et al., 2017). At any rate, the behavior of teachers can vary in terms of the specific, individual characteristics of their students. For example, Waters (2020) points out how students with a lower degree of autonomy tend to mechanically reproduce the learning strategies suggested by their teachers without critically evaluating them.

A further contribution provided by this study concerns the evidence for the importance of peers in the reinforcement of self-efficacy. Until now, studies that dealt with peer support (Orejudo et al., 2020) have only described its sources and its relationships with other support sources but not information about the role it can exert in reinforcing self-efficacy. Our data shows that peers are indeed important, particularly in the 16–18-year-old stage, where adolescents become more independent from their families, and where also, the opinion of peers acquires a more significant weight. This is also the moment of choosing a profession: a point in time in which peers can play a fundamental role by reinforcing elements associated with self-efficacy, such as the selection of professional and educational goals related with music, such as daily practice, and one’s own sense of self-worth. These latter aspects were taken into account in our self-efficacy tool, without ignoring the possible role they might play in certain elements of criticism of musical education, public performance, and the anxiety associated with the latter. Zarza-Alzugaray et al. (2020) have shown how peer support plays a key role in helping boys cope with performance anxiety. A similar idea can be found in the study by Hendricks (2014), who attributed an improvement in girls’ perceived self-efficacy over the course of a 3-day music festival to the support they perceived from peers (among other factors, and only when the level of performance was more non-competitive). Among other concerns, Hewitt (2015) highlights peer context as one of the factors that can exert an influence on the development of self-efficacy – more concretely, self-evaluation. Thus, apart from the role of peers as a general support factor, they can also have an important part in the development of further self-efficacy elements: for example, by providing evaluative feedback.

One of our study’s unexpected results was that we did not find a relationship between social support and self-efficacy in university-age students. There are several possible reasons for this result. Students who have already made progress in their musical career and have opted for a professional future in the field can already count on a considerable amount of social support from their family, from teachers, and from peers in earlier stages. In this context, there would hardly be any difference among these students in terms of the three support sources. Studies providing a contrast with this result are lacking. Hallam et al. (2012) and Perkins et al. (2017) nevertheless point out that music students at university level perceive a greater amount of support from teachers than students at lower academic levels. Such support, however, can be oriented toward other aspects, such as: general wellbeing, the handling of educational stressors, the cultivation of the students’ professional identity, and the upkeep of their motivation to persevere in their musical education. Such support might thus not have a direct relation with the development of self-efficacy, which, most probably, would be affected by other sources such as the students’ own performances, their comparison of themselves with peers, or their mastery of the curricular requirements of the institution of musical learning in which they are enrolled. It is also possible that the scale’s lack of specificity regarding who provides the support – particularly teachers – can reduce the capacity of the tool we used in this study to identify university students’ sources of support. At university level, these students have new teachers (professors) and different teaching/learning conditions; one-on-one classes, particularly with the professor specialized in their instrument, as well as group classes. Thus, the kind of support received from different professors can be thoroughly different. This aspect is identified by other Social Support Scales, such as, for example, Gluska (2011), which differentiates between social support on the part of the instrument teacher compared with that provided by other teachers.

Another complementary assumption that might explain our results could lie in the fact that when we specifically evaluate self-efficacy as an element pertaining to self-regulated learning, the only relationships that emerged were between self-efficacy for learning and self-efficacy for public performance. Students at university level only feel qualified to perform in public in cases where they are able to apply abilities, perceptions, study habits, and learning resources that they feel they have developed in previous stages: periods during which family, teacher, and peer support were indeed relevant, as our data show. This aspect is in accord with Upitis et al. (2017b), who showed that families gradually modified their type of support for their children as they continually improved their ability to self-regulate. It is to be expected that those students who have not sufficiently developed the necessary learning, motivation, and self-regulation abilities to devote themselves to music (McPherson and Zimmerman, 2011; Varela et al., 2016), will eventually abandon their training. This is confirmed by the revealed existence of feedback between self-efficacy and study habits from the onset. It is also well-known that a poor management of performance anxiety becomes a risk factor under which music students might either cease to make academic progress or abandon their studies and their career altogether (Orejudo et al., 2018). At the moment when the student embarks on university-level studies, a poor handling of stage fright will have new consequences (Casanova et al., 2018).

Thus, the main finding that aim to explain our results in university-level students leads us back to consider the central role played by self-efficacy in the self-regulated learning process. We postulate that self-efficacy acts as one of several essential factors in initial stages of behavior (a moment in which the student sets goals and lays out strategies), in the student’s self-evaluation of achievements, in the internal attribution of results, and in planned behavior. Other studies have confirmed the central role of self-efficacy in this model. Waters (2020) shows how students possessing a greater sense of self-efficacy are able to select goals, lay out strategies, and work toward them. Conversely, the strategies deployed by students with low levels of self-efficacy are less adaptive; they perceive less control of the demands made upon them by their environment, as well as increased levels of discomfort and anxiety. Miksza and Tan (2015) found that students with a greater degree of self-efficacy tend to commit themselves more thoroughly to long-term music-related goals; they apply learning strategies that are more elaborate, and, most of all, they succeed in ensuring a greater degree of quality in their practice and learning, thus committing themselves more profoundly to the task. Hewitt (2015) as well as Miksza and Tan (2015) found a direct relationship between self-efficacy, public performance, and self-evaluation of goals: this is another of the key elements in the self-efficacy model, particularly associated with the phase of reviewing one’s actions and reflecting upon them (Panadero and Alonso-Tapia, 2014). Finally, Bonneville-Roussy and Bouffard (2015) found that an individual’s perception of their musical competency is one of the most significant predictors of “deliberate practice,” a type of programmed, goal-oriented practice that is responsible for musical success.

It is thus possible that students with less self-perceived competency for music practice and for public performance will be less devoted to those activities, and that the probabilities of soon abandoning their musical career will increase. This could likewise explain the decrease in support perceived by 16–18-year-old students, who not only end up calling into question their personal value for musical professional activities (associated with self-efficacy), but who are likewise faced with a series of further decisions that have long-term implications for their professional outlook, thereby compelling them to make a choice between a career in music or to choose another field of pursuit. This finding could explain the great relevance acquired by social support in the group of 16–18-year-olds, an age phase in which it would be important for the social support variable to reinforce not only the student’s self-worth as a musician (self-efficacy), but also the goal associated with it: namely, the choice to pursue a musical career. In other words, not only would self-efficacy be implied in this progression (a necessary condition to continue studying music), but also further dimensions of the self-regulated learning model (McPherson and Zimmerman, 2011; McPherson, 2022) which have not been addressed in our study. Indirect support for this is provided by the finding in this 16–18-year-old group: the higher correlation observed between parent and teacher support, was a necessary condition of great value for achieving progress in a musical career and for gaining access to university-level music studies (MacMillan, 2004; Creech, 2009; Upitis et al., 2017b). Upitis et al. (2017b) ascertain that adolescent music students gradually tend to abandon training to the same degree that they start getting involved in other activities which eventually become incompatible, or they start to find less pleasure in music-making.

Although our study reports a number of findings, it also has certain limitations. One lies in the age range, which only included music students who were pursuing a regular studying activity from a certain age on (11 years old). Other stages of musical training including elementary school, music schools, academies, and music education in normal school have not been analyzed herein. Since these are initial stages, it is likely that social support would serve as a highly significant source of motivation. The role of teachers and family is also essential (Upitis et al., 2017a,b). Furthermore, the scales we used in our study do not differentiate in terms of what kind of concrete support the students can perceive. The questionnaires did not differentiate among the kind of family relatives who can provide support: for instance, the gender of the person providing support can be relevant (McPherson, 2009). Our scales do not also differentiate among different types of teachers. Neither were we able to further explore the different types of support each of those sources can provide (Creech, 2009). A final limitation of our study lies in its very nature, self-report, which, as previously mentioned, cannot gather all the different music teacher/professor functions in different levels of music training: thus, it would be necessary or complimentary to apply other methodologies as well.

## Data Availability Statement

The raw data supporting the conclusions of this article will be made available by the authors, without undue reservation.

## Ethics Statement

The studies involving human participants were reviewed and approved by the CEIC Aragón (CEICA) en su reunión del día May 6, 2019, Acta No. 11/2019. Written informed consent to participate in this study was provided by the participants’ legal guardian/next of kin.

## Author Contributions

SO: generation of ideas, literature review, conceptual framework, methodology, data collection, analysis of data, discussion and conclusion, first writing, second writing review, and funding. FZ-A: generation of ideas, literature review, conceptual framework, methodology, data collection, analysis of data, discussion and conclusion, first writing, and second writing review. OC: generation of ideas, literature review, conceptual framework, methodology, data collection, discussion and conclusion, first writing, second writing review, and funding. GM: generation of ideas, conceptual framework, discussion and conclusion, and second writing review. All authors contributed to the article and approved the submitted version.

## Conflict of Interest

The authors declare that the research was conducted in the absence of any commercial or financial relationships that could be construed as a potential conflict of interest.

## Publisher’s Note

All claims expressed in this article are solely those of the authors and do not necessarily represent those of their affiliated organizations, or those of the publisher, the editors and the reviewers. Any product that may be evaluated in this article, or claim that may be made by its manufacturer, is not guaranteed or endorsed by the publisher.

## References

[B1] Baessler J. Schwarcer R. (1996). Evaluación de la autoeficacia: adaptación española de la escala de Autoeficacia General. *Ansiedad y Estrés* 2 1–8.

[B2] Bandura A. (1997). *Self-Efficacy: The Exercise of Control.* New York, NY: Freeman.

[B3] Bandura A. (2006). Toward a psychology of human agency. *Perspect. Psychol. Sci.* 1 164–180. 26151469 10.1111/j.1745-6916.2006.00011.x

[B4] Barlow D. (2000). Unraveling the mysteries of anxiety and its disorders from the perspective of emotion theory. *Am. Psychol.* 55 1247–1263. 11280938 10.1037//0003-066x.55.11.1247

[B5] Bonneville-Roussy A. Bouffard T. (2015). When quantity is not enough: disentangling the roles of practice time, self-regulation and deliberate practice in musical achievement. *Psychol. Music* 43 686–704. 10.1177/0305735614534910

[B6] Byrne B. M. (2010). *Structural Equation Modeling with AMOS. Basic Concepts, Applications, and Programming.* New York, NY: Routledge.

[B7] Caplan G. (1974). *Support Systems and Community Mental Health: Lectures on Concept Development.* New York, NY: Behavioral Publications.

[B8] Casanova O. Zarza-Alzugaray F. J. Orejudo S. (2018). Differences in performance anxiety levels among advanced conservatory students in Spain, according to type of instrument and academic year of enrolment. *Music Educ. Res.* 20 377–389. 10.1080/14613808.2018.1433145

[B9] Cobb S. (1976). Social support as a moderator of life stress. *Psychosom. Med.* 38 300–314. 981490 10.1097/00006842-197609000-00003

[B10] Creech A. (2009). Teacher-pupil-parent triads: a typology of interpersonal interaction in the context of learning a musical instrument. *Music. Sci.* 13 387–413. 10.1177/102986490901300208

[B11] Creech A. Hallam S. (2003). Parent–teacher–pupil interactions in instrumental music tuition: a literature review. *Br. J. Music Educ.* 20 29–44. 10.1017/S0265051702005272

[B12] Davidson J. W. Howe M. J. A. Moore D. G. Sloboda J. A. (1996). The role of parental influences in the development of musical performance. *Br. J. Dev. Psychol.* 14 399–412. 10.1111/j.2044-835X.1996.tb00714.x

[B13] Egilmez H. O. (2015). Pre-service music teachers’ piano performance self-efficacy belief inversely related to musical performance anxiety levels. *Educ. Res. Rev.* 10 2558–2567. 10.5897/ERR2015.2439

[B14] Girgin D. (2020). Motivation, self-efficacy and attitude as predictors of burnout in musical instrument education in fine arts high schools. *Eurasian J. Educ. Res.* 85 93–108. 10.14689/ejer.2020.85.5

[B15] Gluska A. A. (2011). “Skala wsparcia spoécznego uczniów szkó muzycznych [the scale of social support of students of music schools],” in *Badania Naukowe Nad Edukacj Artystyczn i Kulturow [Scientific Studies in Artistic and Cultural Education]*, eds Sacher W. A. Weiner Y. A. (Bielsko-Biała: Y sza zko a Administra ji), 70–92.

[B16] Gruber H. Lehtinen E. Palonen T. Degner S. (2008). Persons in the shadow: assessing the social context of high abilities. *Psychol. Sci.* 50 237–258.

[B17] Hallam S. Rinta T. Varvarigou M. Creech A. Papageorgi I. Gomes T. (2012). The development of practising strategies in young people. *Psychol. Music* 40 652–680. 10.1177/0305735612443868

[B18] Hendricks K. S. (2014). Changes in self-efficacy beliefs over time: contextual influences of gender, rank-based placement, and social support in a competitive orchestra environment. *Psychol. Music* 42 347–365. 10.1177/0305735612471238

[B19] Hewitt M. P. (2015). Self-efficacy, self-evaluation, and music performance of secondary-level band students. *J. Res. Music Educ.* 63 298–313. 10.1177/0022429415595611

[B20] Howe M. J. A. Sloboda J. A. (1991). Young musicians’ accounts of significant influences in their early lives. 1. The family and the musical background. *Br. J. Music Educ.* 8 39–52.

[B21] Jeppsson C. Lindgren M. (2018). Exploring equal opportunities: children’s experiences of the swedish community school of music and arts. *Res. Stud. Music Educ.* 40 191–210. 10.1177/1321103X18773153

[B22] Kurtuldu M. K. Bulut D. (2017). Development of a self-efficacy scale toward piano lessons. *Educ. Sci.* 17 835–857. 10.12738/estp.2017.3.0209

[B23] Lehmann A. C. Kristensen F. (2014). Persons in the shadow” brought to light: parents, teachers, and mentors - how guidance works in the acquisition of musical skills. *Talent Dev. Excell.* 6 54–70.

[B24] MacAfee E. Comeau G. (2020). The impact of the four sources of efficacy on adolescent musicians within a self-modeling intervention. *Contributions Music Educ.* 45 205–236.

[B25] MacMillan J. (2004). Learning the piano: a study of attitudes to parental involvement. *Br. J. Music Educ.* 21 295–311. 10.1017/S0265051704005807

[B26] Margiotta M. (2011). Parental support in the development of young musicians: a teacher’s perspective from a small-scale study of piano students and their parents. *Aust. J. Music Educ.* 1 16–30.

[B27] McCormick J. McPherson G. (2003). The role of self-efficacy in a musical performance examination: an exploratory structural equation analysis. *Psychol. Music* 31 37–51. 10.1177/0305735603031001322

[B28] McPherson G. E. (2009). The role of parents in children’s musical development. *Psychol. Music* 37 91–110. 10.1177/0305735607086049

[B29] McPherson G. E. (2022). “Self-regulated learning music microanalysis,” in *The Oxford Handbook of Music Performance*, ed. McPherson G. E. (New York, NY: Oxford University Press).

[B30] McPherson G. E. Davidson J. W. (2002). Musical practice: mother and child interactions during the first year of learning an instrument. *Music Educ. Res.* 4 141–156. 10.1080/14613800220119822

[B31] McPherson G. E. McCormick J. (2000). The contribution of motivational factors to instrumental performance in a music examination. *Res. Stud. Music Educ.* 15 31–39. 10.1177/1321103X0001500105

[B32] McPherson G. E. McCormick J. (2006). Self-efficacy and music performance. *Psychol. Music* 34 322–336. 10.1177/0305735606064841

[B33] McPherson G. E. Zimmerman B. J. (2011). “Self-regulation of musical learning: a social cognitive perspective on developing performance skills,” in *MENC Handbook of Research on Music Learning: Volume 2: Applications*, eds Colwell R. Webster P. R. (Oxford: Oxford University Press), 130–175. 10.1093/acprof:osobl/9780199754397.003.0004

[B34] McPherson G. E. Davidson J. W. Faulkner R. (2012). *Music in Our Lives: Redefining Musical Development, Ability and Identity.* Oxford: Oxford University Press.

[B35] McPherson G. E. Osborne M. S. Evans P. Miksza P. (2019). Applying self-regulated learning microanalysis to study musicians’ practice. *Psychol. Music* 47 18–32. 10.1177/0305735617731614

[B36] Mieder K. Bugos J. A. (2017). Enhancing self-regulated practice behavior in high school instrumentalists. *Int. J. Music Educ.* 35 578–587. 10.1177/0255761417689921

[B37] Miksza P. Tan L. (2015). Predicting collegiate wind players’ practice efficiency, flow, and self-efficacy for self-regulation: an exploratory study of relationships between teachers’ instruction and students’ practicing. *J. Res. Music Educ.* 63 162–179. 10.1177/0022429415583474

[B38] Miksza P. Blackwell J. Roseth N. E. (2018). Self-regulated music practice: microanalysis as a data collection technique and inspiration for pedagogical intervention. *J. Res. Music Educ.* 66 295–319. 10.1177/0022429418788557

[B39] Moore D. G. Burland K. Davidson J. W. (2003). The social context of musical success: a developmental account. *Br. J. Psychol.* 94 529–549. 10.1348/000712603322503088 14687460

[B40] Nogaj A. A. Ossowski R. (2015). Social support as a mediator for musical achievement. *Pol. Psychol. Bull.* 46 300–308. 10.1515/ppb-2015-0036

[B41] Orejudo S. Candela C. Casanova O. Cuartero L. M. (2020). A social support scale for music students in music schools, academies, and conservatories: an adaptation into Spanish and a factorial invariance study. *Psychol. Music* 10.1177/0305735620968626

[B42] Orejudo S. Zarza F. J. Casanova O. Rodríguez C. Mazas B. (2017). The relation of music performance anxiety (MPA) to optimism, self-efficacy and sensitivity to reward and punishment: testing the theory of personal vulnerability on a sample of Spanish music students. *Psychol. Music* 45 570–583.

[B43] Orejudo S. Zarza-Alzugaray F. J. Casanova O. (2018). Music performance anxiety. Substance use and career abandonment in Spanish music students. *Int. J. Music Educ.* 36 460–472. 10.1177/0255761418763903

[B44] Osborne M. S. McPherson G. E. Miksza P. Evans P. (2020). Using a microanalysis intervention to examine shifts in musicians’ self-regulated learning. *Psychol. Music* 49 972–988. 10.1177/0305735620915265

[B45] Pajares F. Schunk D. H. (2001). “Self-beliefs and school success: Self-efficacy, self- concept, and school achievement,” in *Perception*, eds Ridingand R. Rayner S. (London: Ablex Publishing), 239–266.

[B46] Panadero E. Alonso-Tapia J. (2014). How do students self-regulate? Review of Zimmerman’s cyclical model of self-regulated learning. *Ann. Psychol.* 30 450–462. 10.6018/analesps.30.2.167221

[B47] Papageorgi I. Creech A. Welch G. (2013). Perceived performance anxiety in advanced musicians specializing in different musical genres. *Psychol. Music* 41 18–41. 10.1177/0305735611408995

[B48] Papageorgi I. Haddon E. Creech A. Morton F. Bezenac C. Himonides E. (2010). Institutional culture and learning II: inter-relationships between perceptions of the learning environment and undergraduate musicians’ attitudes to performance. *Music Educ. Res.* 12 427–446. 10.1080/14613808.2010.520432

[B49] Papageorgi I. Hallam S. Welch G. (2007). A conceptual framework for understanding musical performance anxiety. *Res. Stud. Music Educ.* 28 83–107. 10.1177/1321103X070280010207

[B50] Perkins R. Reid H. Araújo L. S. Clark T. Williamon A. (2017). Perceived enablers and barriers to optimal health among music students: a qualitative study in the music conservatoire setting. *Front. Psychol.* 8:968. 10.3389/fpsyg.2017.00968 28701968 PMC5487403

[B51] Randles C. (2011). “What is a good musician?” An analysis of student beliefs. *Arts Educ. Policy Rev.* 112 1–8. 10.1080/10632913.2010.490774

[B52] Randles C. Ballantyne J. (2018). Measuring self-perceptions of creative identity: a cross-cultural comparison of the creative identities of pre-service music teachers in the US and Australia. *Music Educ. Res.* 20 231–241. 10.1080/14613808.2016.1249360

[B53] Randles C. Muhonen S. (2015). Validation and further validation of a measure of creative identity among USA and Finland pre-service music teachers. *Br. J. Music Educ.* 32 51–70. 10.1017/S0265051714000151

[B54] Randles C. Smith G. D. (2012). A first comparison of pre-service music teachers’ identities as creative musicians in the United States and England. *Res. Stud. Music Educ.* 34 173–187. 10.1177/1321103X12464836

[B55] Randles C. Tan L. (2019). Measuring pre-service music teachers’ creative identities: a cross-cultural comparison of the United States and Singapore. *Br. J. Music Educ.* 36 197–210. 10.1017/S0265051719000172

[B56] Ritchie L. Williamon A. (2007). “Measuring self-efficacy in music,” in *Proceedings of the International Symposium on Performance Science 2007*, eds Williamon A. Coimbra D. (Utrecht: Association Européenne des Conservatoires (AEC)), 307–312.

[B57] Ritchie L. Williamon A. (2011). Measuring distinct types of musical self-efficacy. *Psychol. Music* 39 328–344. 10.1177/0305735610374895

[B58] Ritchie L. Williamon A. (2013). Measuring musical self-regulation: linking processes, skills, and beliefs. *J. Educ. Train. Stud.* 1 106–117. 10.11114/jets.v1i1.81

[B59] Ryan K. J. Boulton M. J. O’Neill S. A. Sloboda J. A. (2000). “Perceived social support and children’s participation in music,” in *Proceedings of the 6th International Conference on Music Perception and Cognition*, eds Woods C. Luck G. B. Brochard R. Seddon F. Sloboda J. A. (Keele: Keele University).

[B60] Sarason B. R. Pierce G. R. Sarason I. G. (1990). “Social support: The sense of acceptance and the role of relationships,” in *Social Support: An Interactional View*, eds Sarason B. R. I Sarason G. Pierce G. R. (New York, NY: Wiley), 97–128.

[B61] Schermelleh-Engel K. Moosbrugger H. Müller H. (2003). Evaluating the fit of structural equation models: tests of significance and descriptive goodness-of-fit measures. *Methods Psychol. Res. Online* 8 23–74.

[B62] Schwarzer R. Jerusalem M. (2010). The general self-efficacy scale (GSE). *Anxiety Stress Coping* 12 329–345.

[B63] Sherer M. Adams C. H. (1983). Construct validation of the self-efficacy scale. *Psychol. Rep.* 53 143–148. 10.2466/pr0.1983.53.3.899

[B64] Sichivitsa V. O. (2007). The influences of parents, teachers, peers and other factors on students’ motivation in music. *Res. Stud. Music Educ.* 29 55–67. 10.1177/1321103X07087568

[B65] Upitis R. Abrami P. C. Varela W. King M. Brook J. (2017c). Student experiences with studio instruction. *Music Educ. Res.* 19 410–437. 10.1080/14613808.2016.1202221

[B66] Upitis R. Abrami P. C. Brook J. Boese K. King M. (2017a). Characteristics of independent music teachers. *Music Educ. Res.* 19 169–194. 10.1080/14613808.2016.1204277

[B67] Upitis R. Abrami P. C. Brook J. King M. (2017b). Parental involvement in children’s independent music lessons. *Music Educ. Res.* 19 74–98. 10.1080/14613808.2016.1202220

[B68] Varela W. Abrami P. C. Upitis R. (2016). Self-regulation and music learning: a systematic review. *Psychol. Music* 44 55–74. 10.1177/0305735614554639

[B69] Waters M. (2020). Perceptions of playing-related Discomfort/Pain among tertiary string students: a thematic analysis. *Music Educ. Res.* 22 257–269. 10.1080/14613808.2020.1765154

[B70] Watson K. (2010). The effects of aural versus notated instructional materials on achievement and self-efficacy in jazz improvisation. *J. Res. Music Educ.* 58 240–259. 10.1177/0022429410377115

[B71] Zarza-Alzugaray F. J. Casanova O. McPherson G. E. Orejudo S. (2020). Music self-efficacy for performance: an explanatory model based on social support. *Front. Psychol.* 11:1249. 10.3389/fpsyg.2020.01249 32670146 PMC7330084

[B72] Zelenak M. S. (2015). Measuring the sources of self-efficacy among secondary school music students. *J. Res. Music Educ.* 62 389–404. 10.1177/0022429414555018

[B73] Zimmerman B. J. (2000). Self-Efficacy: an essential motive to learn. *Contemp. Educ. Psychol.* 25 82–91. 10.1006/ceps.1999.1016 10620383

